# Efficacy of Pyrotinib in HER2-Overexpressing Salivary Duct Carcinoma With Lung Metastasis: A Case Report

**DOI:** 10.3389/fonc.2020.559057

**Published:** 2020-10-02

**Authors:** Zi-yan Yang, Jia-huan Huang, Bo Chen, Chun-wei Xu, Lei Lei, Xiao-jia Wang, Mei-yu Fang

**Affiliations:** ^1^Department of Breast Oncology, Cancer Hospital of the University of Chinese Academy of Sciences (Zhejiang Cancer Hospital), Hangzhou, China; ^2^Institute of Cancer and Basic Medicine (ICBM), Chinese Academy of Sciences, Hangzhou, China; ^3^Department of Oncology, Zhejiang Provincial People's Hospital, People's Hospital of Hangzhou Medical College, Hangzhou, China; ^4^Department of Pathology, Cancer Hospital of the University of Chinese Academy of Sciences (Zhejiang Cancer Hospital), Hangzhou, China; ^5^Department of Respiratory, Affiliated Jinling Hospital, Medical School of Nanjing University, Nanjing, China; ^6^Department of General Oncology, Key Laboratory of Head and Neck Cancer Translational Research of Zhejiang Province, Institute of Cancer Research and Basic Medical Sciences of Chinese Academy of Sciences, Cancer Hospital of the University of Chinese Academy of Sciences, Hangzhou, China

**Keywords:** salivary duct carcinoma, tyrosine kinase inhibitor, pyrotinib, HER2 positive, targeted

## Abstract

**Background:** Salivary duct carcinoma (SDC), an aggressive and rare malignancy with poor prognosis, is mostly associated with the overexpression of the androgen receptor (AR) and human epidermal growth factor receptor 2 (HER2). However, limited data are available for the targeting of both HER2 and AR in advanced/metastatic SDC.

**Case Presentation:** A 62-year-old man with advanced SDC accompanied by lung and lymph node metastasis showed disease progression after two lines of chemotherapy and endocrine therapy. Metastatic lesions from the lung biopsy were obtained, and immunohistochemistry (IHC) indicated the overexpression of AR and HER2 (3+). The patient was administered pyrotinib (a pan-ErbB receptor tyrosine kinase inhibitor) and bicalutamide (an androgen receptor antagonist) as a third-line treatment. During the ten months of follow-up, a durable partial response was achieved with this combination.

**Conclusions:** This is the first clinical study to report the successful application of pyrotinib in a patient with advanced SDC. We recommend that pyrotinib and bicalutamide be used as salvage therapy for AR and HER2-positive advanced metastases in SDC, given the favorable response and clinical benefit.

## Introduction

Salivary duct carcinoma (SDC) is a rare malignancy, categorized into 22 subtypes with clearly different histological, genetic, and clinical characteristics (1). SDC is an uncommon type of salivary gland cancer (SGC), making up almost 1–3% of all SGCs (2). It is a disease with aggressive behavior and a tendency to recur and metastasize, usually in the lymph node, lungs, bone, or other visceral organs (3). Since SDC progresses rapidly, most patients die within three years of diagnosis (4). For resectable tumors, surgery with or without postoperative radiotherapy is the main treatment. However, in the case of metastatic SDC, systemic chemotherapy has had disappointing outcomes (5). Importantly, the diagnosis and treatment of different subtypes of SGC depend on clinicopathology, emphasizing the importance of pathological examination. HER2 overexpression, which occurs in 29–46% of patients with SDC, is associated with poor prognosis (6). The characteristics of SDC are similar to those of breast cancer in terms of morphology and gene expression patterns. HER2-targeted therapy has been established as the standard therapy for HER2-positive breast cancer. This suggests that HER2-targeted therapy may be an ideal choice for HER2-overexpressing SDC. Pyrotinib is an irreversible pan-ErbB receptor tyrosine kinase inhibitor (TKI) with anti-EGFR/HER1, HER2, and HER4 activities. In China, it has proven to be successful as a second-line treatment or post-treatment therapy in advanced or metastatic HER2-positive breast cancer and has shown a strong anti-tumor effect in clinical trials.

Here, we describe a case of a patient with overexpression of HER2 and advanced SDC who attained partial remission after third-line treatment with pyrotinib.

## Case Report

**Figure 1:**
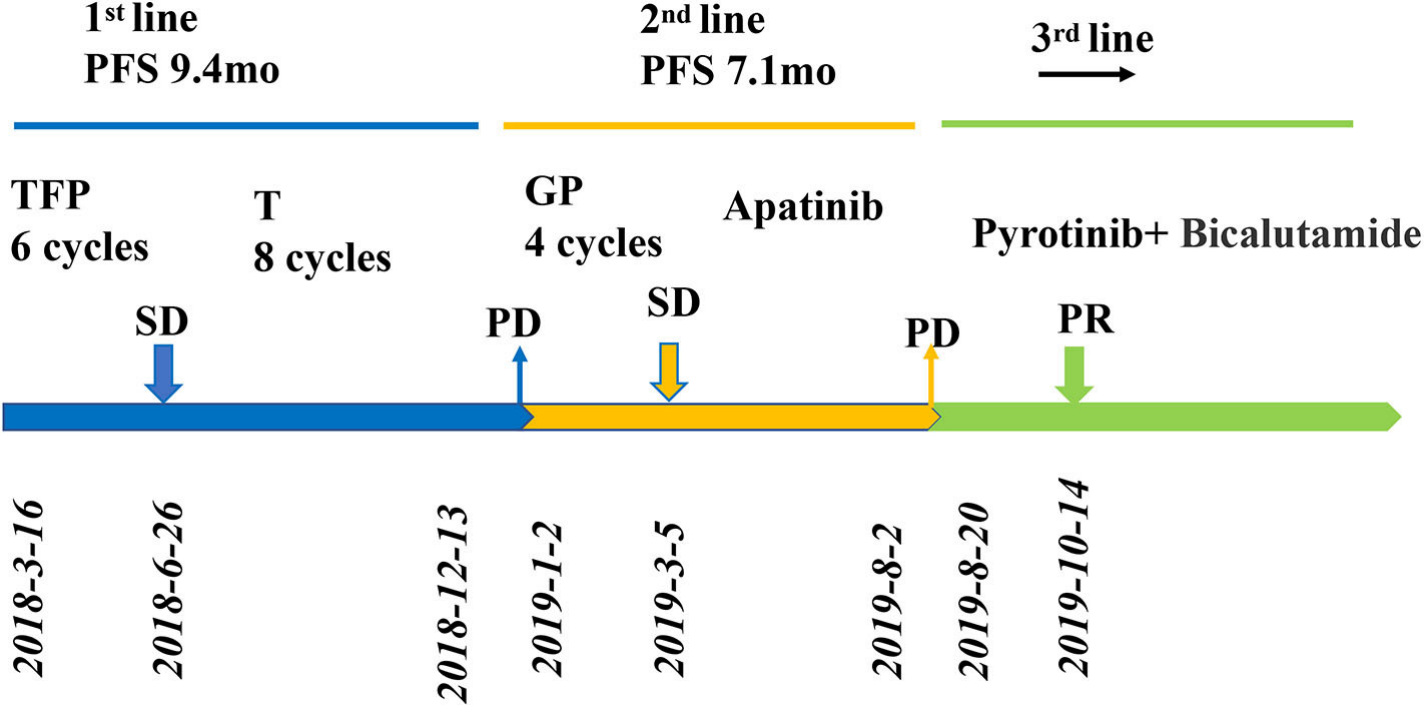
Review of treatment process (from March 2018 to May 2020). Treatment process and effect evaluation of the patient. PFS, progression-free survival; TFP, docetaxel, fluorouracil, cisplatin; T, docetaxel; GP, gemcitabine/cisplatin; PR, partial response; SD, stable disease; PD, progressive disease; mo, months.

A 62-year-old man presented with a right parotid mass in February 2018. He complained of pain but denied fever and weight loss. The patient was referred to our hospital for further examination, and a follow-up computed tomography (CT) scan showed a mass with a diameter of 2.3 cm × 1.8 cm on the right parotid with dual cervical, supraclavicular lymph nodes, and lung metastasis. No other remote abnormal lesions were found in the body. Fine-needle aspiration biopsy of the right parotid mass and neck lymph node (right side) confirmed SDC with a metastatic lymph node, and immunohistochemistry (IHC) results were positive for the expression of CK18, CK5/6, EMA, AR, and Ki-67, but negative for that of P40 and EBER. Based on the CT scan findings and pathological and molecular results, the patient was diagnosed with SDC along with metastasis in the lymph nodes and lung. Based on the above pathological results and clinical description, combined with the 8th edition of the American Joint Committee on Cancer (AJCC), this patients tumor (T) lymph node (N) metastasis (M) staging is cT2N2M1 (stage IV).The patient had a family history of cancer and addiction to alcohol, but denied a history of hypertension, diabetes mellitus, or coronary heart disease, or addiction to tobacco. On admission, the patient appeared to be in a good clinical condition and the performance status (PS) was 0. Routine blood tests, such as complete blood count (CBC) and biochemical indices were all within normal ranges, an electrocardiogram (ECG) showed sinus rhythm, echocardiography displayed slight mitral regurgitation, and left ventricular ejection fraction was (LVEF) was 76%. There was no fever or difficulty in breathing. There were no relevant comorbidities and no concomitant medications were administered. The patient was an office employee who had no history of carcinoma or exposure to environmental risk factors. Figure 1 illustrates the complete treatment procedure and the corresponding changes in the lesions on CT.

**Figure 2:**
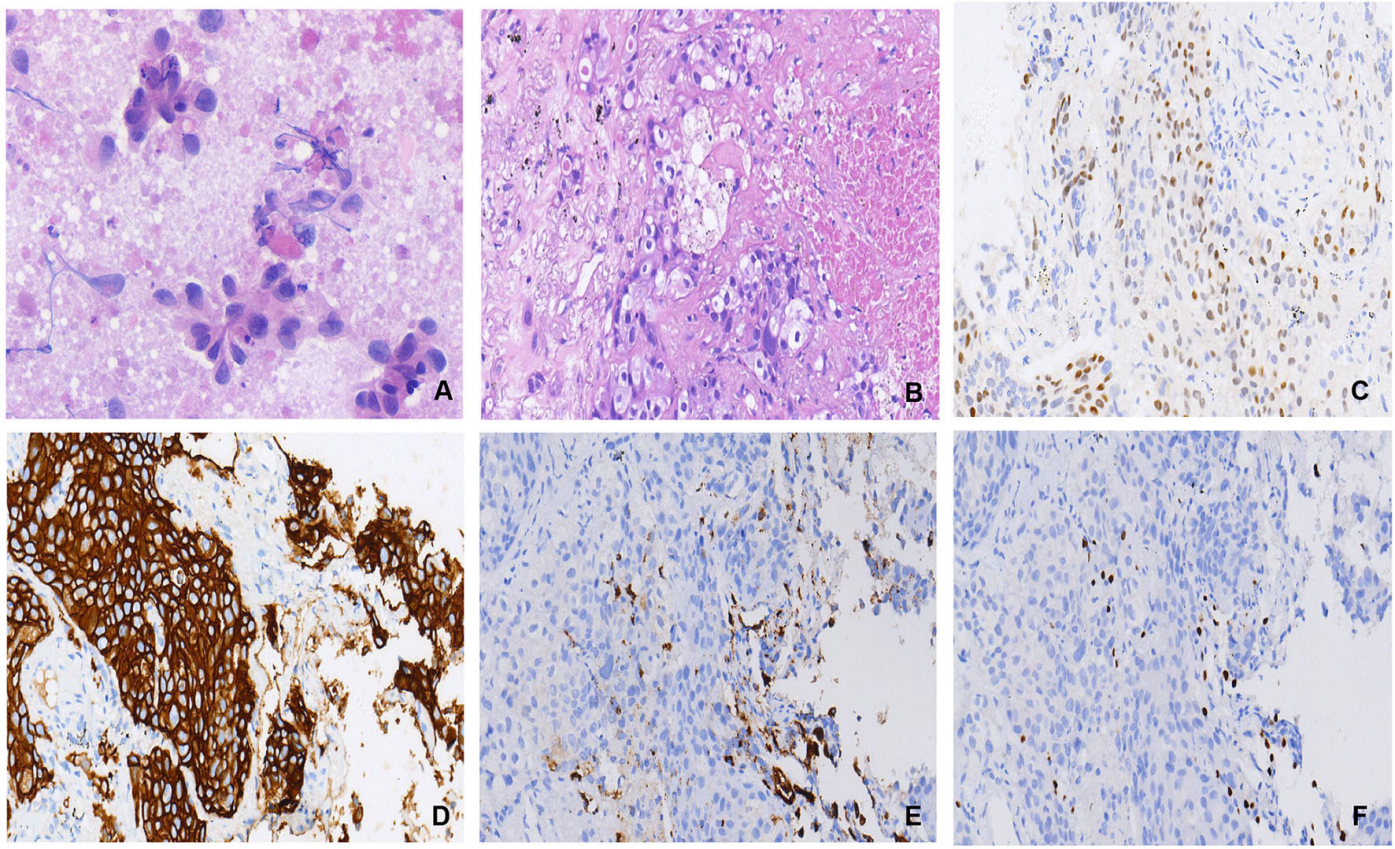
**(A)** Cytological features of fine-needle aspiration biopsy of the right parotid mass: few poorly differentiated carcinomas with necrosis (×20 hematoxylin/eosin). **(B)** Histopathological features of CT-guided lung mass biopsy: cancer cells in the lung tissue grow invasively, with local necrosis (×20 hematoxylin/eosin) **(C)** Androgen receptor (AR) (+,40%) (×20 hematoxylin/eosin) **(D)** The human epidermal growth factor receptor-2(HER2) demonstrated 3+ reactivity to show positive (×20 hematoxylin/eosin) **(E)** NapsinA: cancer cells were negative, while remaining alveolar epithelial cells were positive (×20 hematoxylin/eosin) **(F)** TTF1: cancer cells were negative, while remaining alveolar epithelial cells were positive (×20 hematoxylin/eosin).

Radical surgery was not available. The patient was initially treated with TFP chemotherapy (46 mg cisplatin, 7,600 mg fluorouracil, and 140 mg docetaxel). CT examinations showed stable disease after two cycles in both the lungs and lymph nodes. After six cycles, the regimen was changed with docetaxel as maintenance therapy until December 2018, when a chest CT scan showed progressive lesions in the lung. At the end of the treatment, the neck lesions had receded, and the symptoms were completely relieved. After the six-month-long docetaxel maintenance treatment, the patient presented to the outpatient clinic for cough and fever evaluation in January 2019. The results of the chest CT scan showed recurrent disease in the neck lymph nodes and lung metastasis. Subsequently, we performed IHC on the primary lesion, which showed that the HER2 receptor was overexpressed (+3). The patient underwent a new second-line treatment with four cycles of chemotherapy (1.9 g gemcitabine and 45 mg cisplatin) followed by apatinib as maintenance therapy. However, seven months later it was found that the disease had progressed. The pulmonary metastases had increased dramatically as evaluated by CT scan (August, 2019), according to the Response Evaluation Criteria in Solid Tumors (RECIST) version 1.1. In August 2019, CT-guided lung mass biopsy was performed for further histopathological diagnosis. HE staining combined with IHC of the biopsy tissues (Figure 2; positive for AR [40%], HER2 [3+], Ki-67 [80%], CK5/6, and EGFR, but negative for ER, PR, S-100, CD117, P53, TTF, and napsinA) confirmed that the parotid adenocarcinoma had metastasized to the lung.

**Figure 3:**
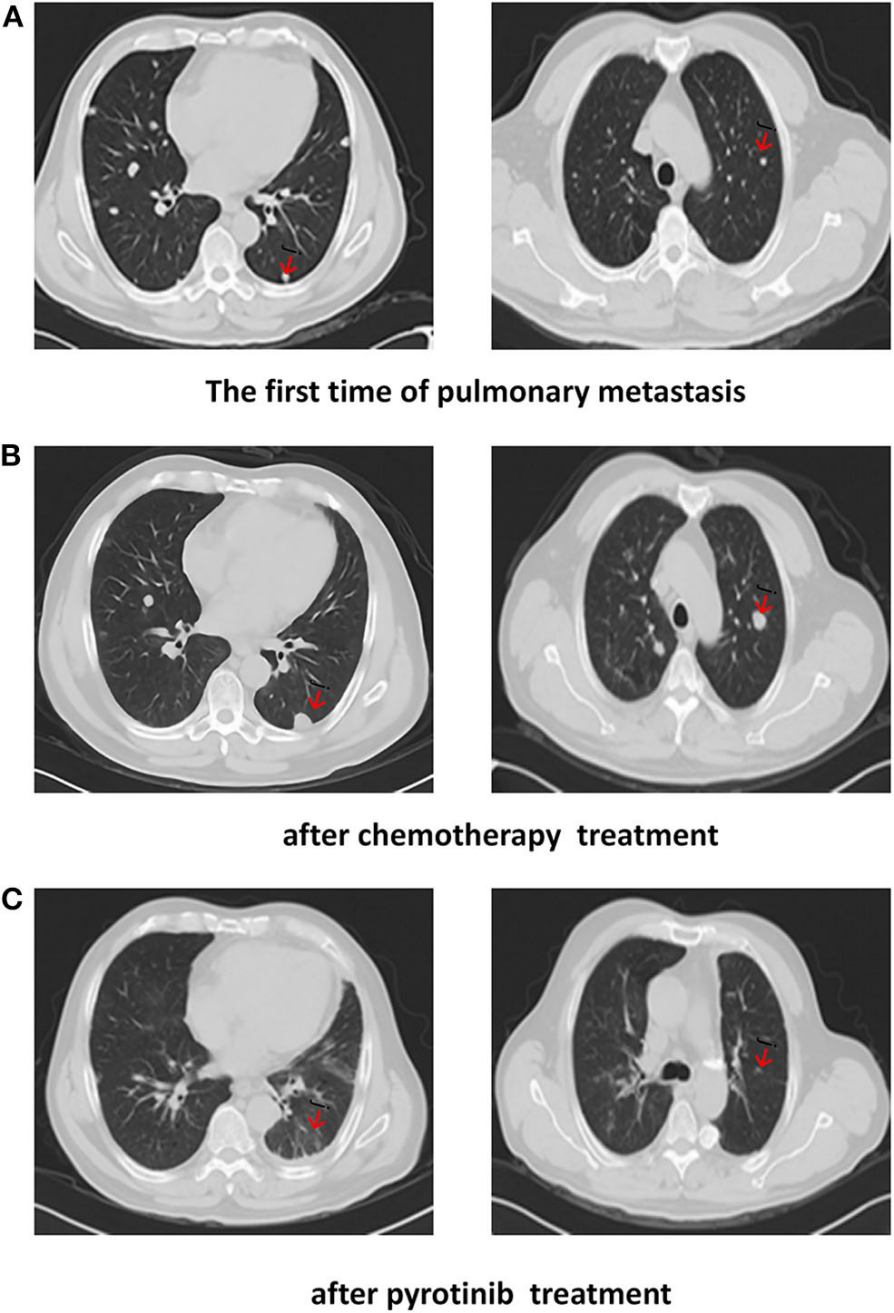
CT scans show: **(A)** The first time of pulmonary metastasis; **(B)** after chemotherapy treatment; **(C)** CT of the chest showed a complete response after months of pyrotinib treatment.

Based on the clinical history and pathological findings of the lung tissue, primary lung adenocarcinoma was excluded. The patient had a parotid ductal carcinoma with lung metastasis. It was determined that the patient would benefit from anti-HER2 therapy and endocrine therapy due to the overexpression of AR (+, 40%) and Her2 (3+). Thus, the patient received pyrotinib (400 mg QD) and bicalutamide (50 mg QD) from July 2019 to October 2019. The skin lesions on the neck disappeared after two cycles. CT scans performed after 3 months of treatment showed that the target pulmonary metastatic lesions completely disappeared, and the remaining lesions were significantly reduced, indicating a PR with a >30% decrease in the sum of the diameter compared with the previous examinations (Figure 3). The patient showed a significantly improved performance status and symptoms and never complained of cough and thoracalgia. The patient showed good compliance, took pyrotinib according to the instructions, and came to the hospital for review every 2 cycles. He tolerated the treatment with pyrotinib well. Grade 1 diarrhea with no additional adverse events was observed during the treatment with pyrotinib; this improved soon after the symptomatic treatment. The period of progression-free survival (PFS) was over 10.2 months.

Until the submission of this case draft, the patient has survived for over 27 months and now continues to receive the combination treatment of pyrotinib and bicalutamide; at the time of writing this case, the patient's condition was stable.

This study was approved by the Ethics Committee of Zhejiang Cancer Hospital, Hangzhou, China. Written informed consent was obtained from the participant for the publication of this case report and any potentially identifying images or information.

## Discussion

The treatment of SDC is different in various studies; however, most SDCs are surgically removed from the primary site by dissection, and the patient then undergoes adjuvant treatment (7). Traditionally, cyclophosphamide, doxorubicin, and cisplatin have been used in the treatment of recurrent or metastatic SGC (8), whereas HER-2 targeted therapy and androgen deprivation therapy have been documented in clinical studies of SDC. Our case study shows the excellent results of using pyrotinib in combination with bicalutamide in treating a patient with SDC who had lung metastasis with HER2- and AR-positivity.

SDC is a rare and highly aggressive type of salivary gland carcinoma that is associated with a high rate of local recurrence and metastasis; thus, treatment options have been to date rather limited. The response to standard management of the disease through systemic chemotherapy for metastatic SDC has been disappointing. SDC is similar to high-grade ductal breast carcinoma, both in morphologic and molecular features. In particular, several characteristic findings have been exploited wherein one-third of the patients with SDC demonstrated HER2 protein expression and/or ERBB2 amplification; thus, the treatment is guided by what is known about breast cancer with HER2 overexpression. In several studies the activity of various anti-HER2 drugs, alone or with chemotherapy, in individual patients with HER2-positive SDC has been investigated (9, 10). In a multi-center, nonrandom, open, multi-cohort basket trial of the Mypathway study (11), five patients with HER2-amplified SDC—four of which were PR, with an objective response rate (ORR) of 80% were effectively treated with trastuzumab plus pertuzumab. This is evidence that targeted therapy is effective for SDC. However, in another study, Chiosea et al. (12) pointed out that trastuzumab is used to treat patients with HER-2 overexpressing breast cancer, accompanied by PIK3CA mutation or PTEN deletion. The efficacy may be limited. Despite the progress in targeted therapy for SDC, it has not been standardized or clinically validated, and the inevitable drug resistance requires irreversible drug options.

In the present study, out of consideration of cost, toxic reactions, and ease of use, a new-generation, targeted drug, pyrotinib, was given to the patient, and this drug was still effective after two lines of treatment. Pyrotinib is an oral, irreversible, pan-ErbB TKI that potently blocks downstream pathways that are activated by EGFR/HER1, HER2, and HER4. Preclinical and phase I studies demonstrated that multiple HER receptors can inhibit HER2 overexpression both *in vivo* and *in vitro* (13). In a retrospective phase II study that included 128 patients with HER2-positive metastatic breast cancer treated with pyrotinib in combination with capecitabine, the ORR of the patients significantly increased (78.5 vs. 57.1%) and PFS was prolonged (18.1 vs. 7.0 months) compared to those treated with lapatinib in combination with capecitabine (14, 15). Furthermore, a phase III study (PHOEBE) showed that pyrotinib plus capecitabine significantly prolonged PFS by 5.7 months (12.5 vs. 6.8 months) compared with lapatinib plus capecitabine. Accordingly, pyrotinib was approved in China as a second-line standard regimen for metastatic HER2-positive breast cancer (16). In a clinical study in which the effectiveness of three anti-HER2 drugs—afatinib, T-DM1, and pyrotinib—were compared, it was found that all were able to significantly block HER2 downstream signaling pathways. Of the three drugs, pyrotinib had the best anti-tumor activity at a reasonable dose (17).

According to relevant literature (18, 19), pyrotinib also has an excellent effect on other HER2-positive overexpressing solid tumors. Gastric and breast cancers, especially the HER2-positive type, have a poor prognosis. For patients who have developed resistance to trastuzumab, pyrotinib is a promising new agent. In the case that we report here, the tumor shrank markedly with the combination therapy of bicalutamide and pyrotinib.

Good results have been obtained from the use of bicalutamide as an androgen deprivation drug, together with nilotinib. In one study in which 98% of SDC patients had AR expression, the researchers treated the AR-positive patients with androgen receptor antagonist, and the results showed that it was effective in ~50% of the patients (20). Patients with high AR expression have a better prognosis than AR-negative patients, whose 2-year disease-free survival (DFS) had significantly increased (59.0 vs. 28.6%) (21). Although the mechanism is not clear, it may be due to the activation of the downstream gene phosphatidylinositol-3-kinase (PI3K) of HER-2, which promotes the combination of AR and androgens downstream of the PI3K/mitogen-activated protein kinases (MAPK) pathway to form AR homodimers transferred to the nucleus. Therefore, AR homodimers binds to the *cis*-acting element of the upstream deoxyribonucleic acid (DNA) of the promoter and interacts with other co-activators to regulate the transcription function of genes, thereby inhibiting the proliferation of SDC tumors. In general, AR can regulate the transcription of multiple effector genes by binding to DNA or interacting with other transcription factors, and can inhibit the growth, differentiation, and survival of tumor cells.

As shown in this case study, HER2 amplification and/or HER2 overexpression testing should be considered in the diagnosis of advanced SDC, given that anti-HER2 therapy, such as pyrotinib, can promote good clinical outcomes. Additionally, in this case, the patient expressed AR (78–96% of the cases), which is a defining feature of SDC (22). However, in this study, the patient was not select for conventional targeted therapy of trastuzumab, so the effectiveness cannot be compared with targeted therapy with pyrotinib. This is the limitation of treatment in this case. For advanced tumors, treatment strategies need to be individualized, and a combination of IHC and imaging diagnosis can further confirm the diagnosis and prognosis as well as guide treatment. Multiple studies have shown that AR and HER-2 may be potential biomarkers of SDC, providing a molecular basis for individual stratification of SDC and new targeted multi-drug combination therapy.

## Conclusion

To the best of our knowledge, this is the first case report describing the successful and efficacious use of pyrotinib combined with bicalutamide in an SDC patient with HER2 and AR overexpression. Because a favorable response to this therapy has been observed, other SGC patients with HER2 expression should be maintained on an HER2 blockade even after second-line progression.

## Ethics Statement

Written informed consent was obtained from the individual(s) for the publication of any potentially identifiable images or data included in this article.

## Author Contributions

All authors have read and approved the final manuscript. ZY and JH wrote the manuscript. BC undertook the pathological diagnosis. MF, LL, and XW carried out the clinical management of the patient. CX collected the patient's clinical data and analyzed the data.

## Conflict of Interest

The authors declare that the research was conducted in the absence of any commercial or financial relationships that could be construed as a potential conflict of interest.
